# Performance of Low-Temperature Bleaching Techniques for Cotton Fabrics Using Hydrogen Peroxide Activators

**DOI:** 10.1021/acsomega.5c02605

**Published:** 2025-06-27

**Authors:** Letícia Küster, Bruna Porto, Catia Rosana de Aguiar, Miguel Angelo Granato

**Affiliations:** Postgraduate Program in Textile Engineering, Federal University of Santa Catarina, Blumenau Campus, Engenheiro Udo Deeke St, 485, Salto do Norte, Blumenau, Santa Catarina 89065-100, Brazil

## Abstract

Cotton fibers contain impurities that must be removed before dyeing/printing. Traditional hydrogen peroxide bleaching is energy- and time-intensive, requiring near-boiling temperatures and over 60 min. This study evaluates triacetin, peracetic acid, and performic acid as hydrogen peroxide activators to lower the temperature and process time in cotton bleaching. The best results were achieved with 0.9 g·L^–1^ triacetin and 1.0 g·L^–1^ peracetic acid at 80 °C for 30 min, reaching 61.1 Berger whiteness. Subsequent tests revealed a slight color difference in dyeings with triacetin (0.5 Δ*E* CMC2:1) and high dye affinity (103% tinctorial strength) compared to conventionally bleached samples. Lighter colors showed a greater bleaching influence. This result is 97% similar to that of conventional bleaching, suggesting a promising alternative for sustainable textile production. This approach stands out for its ability to reduce processing temperatures and times, resulting in lower energy consumption and greater efficiency while maintaining nearly the same fabric whiteness and dyeing quality as conventional methods. It also provides insights into color sensitivity during bleaching.

## Graphical abstract

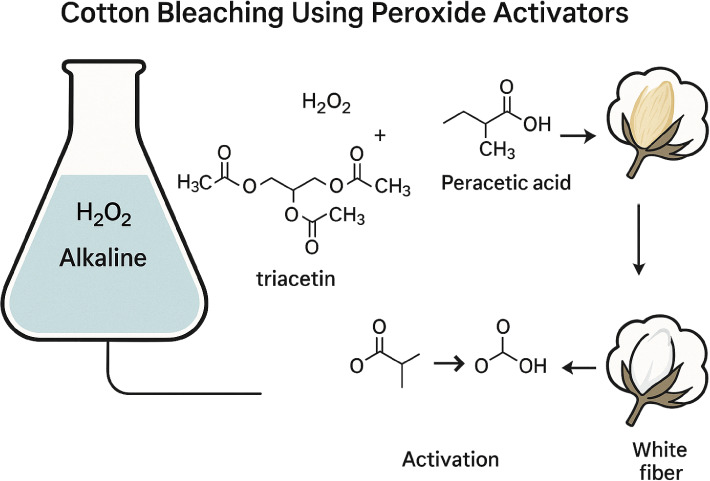

## 1 Introduction

Raw cotton contains natural impurities that affect the whiteness of pure cellulose. Consequently, cotton yarn as it exits the spinner invariably has a soiled or grayish color. When such yarn is woven, it becomes further contaminated with substances introduced during the sizing of the warps. The process of removing these natural coloring matters and add-ons during the previous state of manufacturing is called scouring and bleaching.^1^ There are several bleaching processes for textiles such as (a) oxidative,^2,3^ (b) reductive,^4^ and (c) enzymatic.^5,6^ The objective of bleaching is to eliminate natural coloration for the following steps such as dyeing or printing or to achieve full whiteness.^7^

Bleach activators are organic acid derivatives, such as amides and esters, which can produce organic peracids at lower temperatures in situ due to a nucleophilic substitution of the perhydroxyl anion on the amide or ester groups.^8,9^ The H_2_O_2_/bleach activator bleaching process is a two-step reaction for cotton bleaching. The first step is the formation of peracids due to a nucleophilic reaction of HOO– with bleach activators, and the second is the bleaching reaction caused by the resulting peracids.^10^ Furthermore, according to Wang, the performance of the H_2_O_2_ system depends not only on chemical reactions but also on electrostatic interactions between the activators and the cotton surface.^11^

Some examples of bleach activators are tetraacetylethylenediamine (TAED) and sodium nonanoyloxybenzenesulfonate (NOBS). NOBS has gained acceptance as a bleach precursor in the United States and Japan, while TAED is the main activator used in Europe.^12^ These bleach activators react with hydrogen peroxide in aqueous solution to form peroxy acids, which are more potent bleaches than hydrogen peroxide at lower temperatures (<60 °C). However, they are too unstable to be stored in their active form and must therefore be generated in situ.^13^ Another example of a bleach activator for H_2_O_2_ cotton bleaching is tetraacetylhydrazine (TH). TH has two acetyl groups that can convert into peracetic acid in the H_2_O_2_/TH bleaching system. Liu et al. achieved a performance with the H_2_O_2_/TH system similar to that of the H_2_O_2_/TAED system with minimal damage to bursting strength. TH has better water solubility than TAED, allowing it to react more easily with HOO– to produce peracetic acid.^14^ Additionally, Huang et al. introduced acetylated starch (AS) as a biodegradable and ecofriendly bleach activator. Their system achieved a whiteness index of 72 (CIE WI) at just 70 °C in 60 min using 6 g·L^–1^ of AS, reinforcing the potential of alternative activators for low-temperature bleaching of cotton fabrics.^15^

Triacetin (glycerol triacetate (GT), Figure 1a) can react with HOO–, decomposed by hydrogen peroxide (H_2_O_2_), to generate peracetic acid (PAA; Figure 1b). This reaction, in turn, facilitates the decomposition of H_2_O_2_ and the production of hydroxyl radicals (HO•) at lower temperatures and under alkaline conditions, making it more efficient compared with other activators. In this context, both PAA and HO• are believed to play significant roles in the H_2_O_2_/GT bleaching system (Figure 2). Despite its promising attributes, triacetin has been identified as a cotton bleaching activator in the literature only once,^16^ indicating that its utilization remains largely unexplored. Zhou et al. obtained acceptable whiteness to bleach cotton knitted fabrics by using 2.2 g·L^–1^ of triacetin at 60 °C for 60 min. In this study, we investigated the influence of temperature, processing time, and the type and concentration of auxiliary chemicals—specifically evaluating the performance of 0.9 g/L glycerol triacetate (GT)—to achieve effective bleaching suitable for light-hue dyeing.^16^ Compared to the work of Zhou et al., where 2.2 g·L^–1^ of triacetin was required at 60 °C for 60 min to reach acceptable whiteness in cotton knits,^16^ our approach demonstrates the potential for achieving similar results under milder conditions and with lower chemical usage. Furthermore, a complete characterization of the bleached samples was performed to assess the impact of the proposed bleaching process on their properties, including parameters such as hydrophilicity, capillarity, and morphology, which were not examined in a previous study. It is worth noting that this work is directly designed for the industrial application of the bleaching process, whose final objective is to obtain the best degree of whiteness for subsequent dyeing with light colors, thus reducing process times and energy consumption.

**Figure 1:**
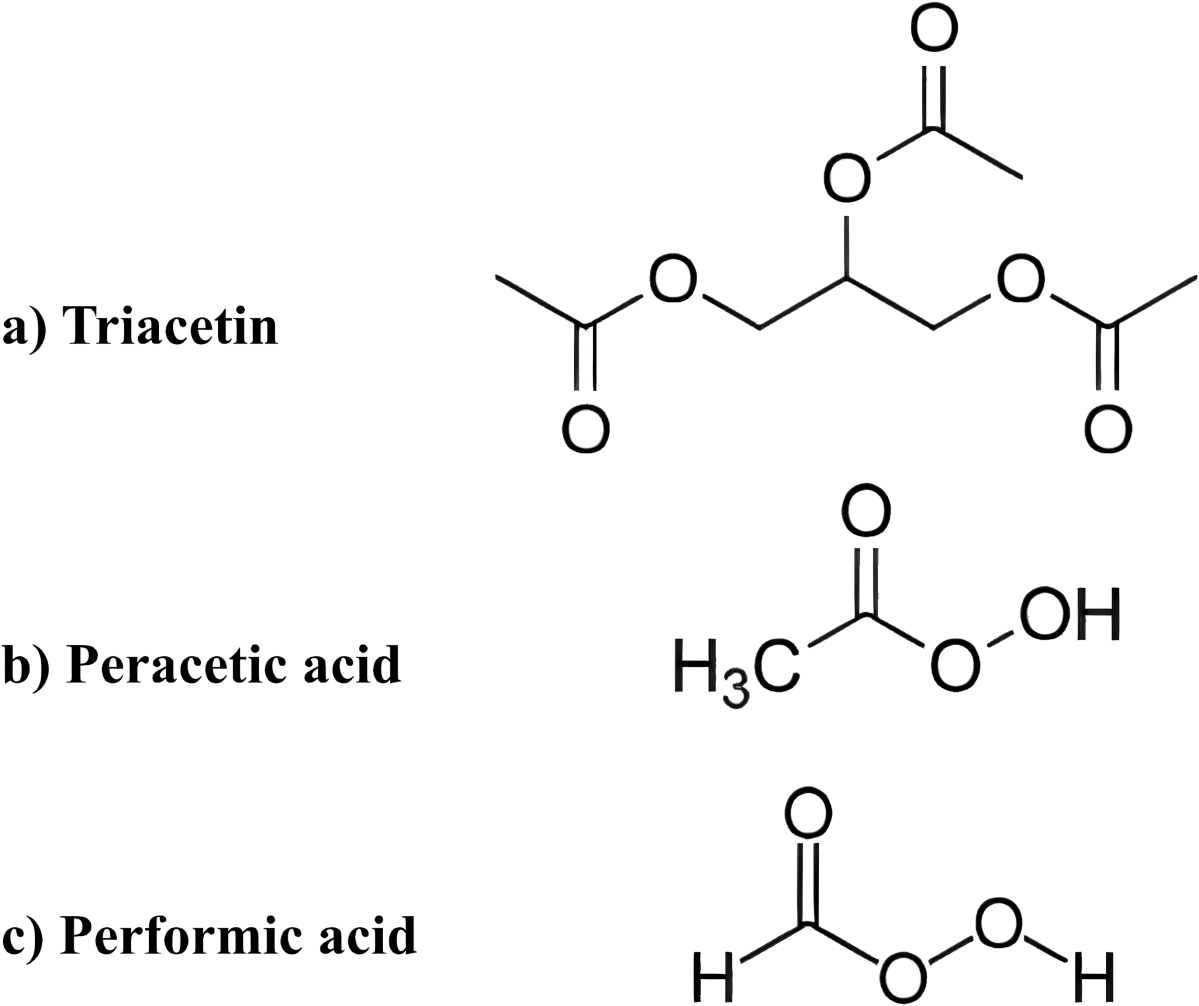
Chemical structure of triacetin (a), peracetic acid (b), and performic acid (c).

**Figure 2:**
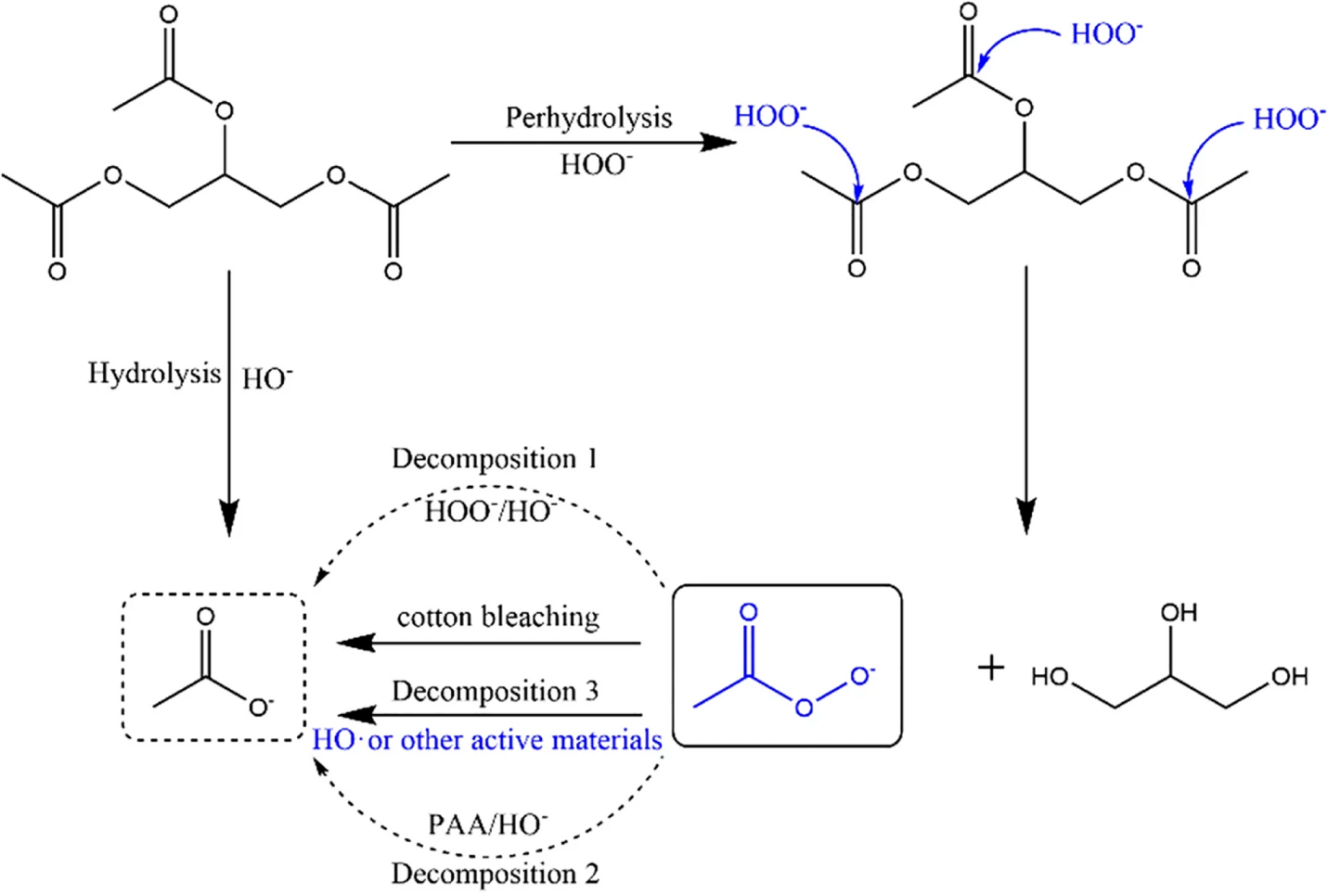
Bleaching routes for the H_2_O_2_/GT system (reprinted with permission^16^ —Springer Nature).

## 2 Materials and Methods

The experiments were carried out in the laboratories of Brandili Têxtil in Apina, located in the state of Santa Catarina, Brazil. The company’s management personnel kindly provided equipment and raw materials.

### 2.1 Materials

#### 2.1.1 Chemicals

The following chemicals were used to bleach the raw cotton: ethoxylated C12–C16 fatty alcohol as the dispersing agent, peracetic and performic acids, and triacetin as peroxide activators, industrial grade, provided by Hanier Especialidades Químicas Ltd. Hydrogen peroxide and sodium hydroxide were provided by BSC Química Ltd. Etidronic acid (1-hydroxyethane-1,1-diphosphonic acid, HDEPA) was used as a chelating agent and supplied by Hanier Especialidades Químicas Ltd.

For dyeing the bleached samples, a light pink shade was chosen, obtained by mixing two reactive dyes: C.I. Reactive Yellow 145 (0.001% owf) and C.I. Reactive Red 195 (0.016% owf) were both supplied by Colortex Brasil.

#### 2.1.2 Substrate

The substrate was produced from yarns made with 100% combed cotton fibers, with a 30/1 Ne title, at the company’s knitting plant, located in Rodeio, Santa Catarina. It has the specification of “combed knit”, with a grammage of 175 g·m^–2^. According to the supplier, the provided fibers had already undergone desizing and scouring stages to remove impurities, thus being suitable for application in bleaching studies.

### 2.2 Experiments

#### 2.2.1 Design of Experiments (DOE) for the Low-Temperature Bleaching Process

In industry, bleaching at low temperatures (75 °C) is not commonly applied for light or clean colors due to the insufficient whiteness achieved, rendering the process unsuitable. In such cases, conventional bleaching at high temperatures (100 °C) continues to be the standard practice. To explore alternatives that prioritize low-temperature processes for industrial applications, adjustments to variables, such as process duration, auxiliary chemicals, and their concentrations, were investigated.

Table 1 outlines the process conditions for both conventional high-temperature bleaching (95–98 °C) and low-temperature bleaching (75 °C) employed by the company Brandili Têxtil. The objective was to identify optimal conditions at lower temperatures that could achieve the desired degree of whiteness, enabling subsequent dyeing with light colors.

**Table 1:** Conditions of the Bleaching Processes

Condition	Bleaching @95–98 °C	Bleaching @75 °C
Wetting agent (% owf)	1.2	1.0
Chelating agent (% owf)	0.27	0.38
Sodium hydroxide (% owf)	2.5	3.3
Hydrogen peroxide (% owf)	2.8	3.0
Triacetin (g·L^–1^)	0.0	0.9
pH	11	11
Process time (min)	15	15

The bleaching process parameters were determined by using an experimental design based on the current formulation applied to medium and dark colors, adapted for low-temperature conditions (75 °C). The experiments were divided into two blocks to isolate the time variable:^17^ (i) tests conducted at 75 °C for 15 min and (ii) tests conducted at 75 °C for 30 min.

Table 2 lists the chemical products and their fixed concentrations, while Table 3 presents the varying amounts of hydrogen peroxide activators used across all experiments (1 to 28). The initial experiments (1 to 17) were conducted at 75 °C, and based on their results, new tests (18 to 28) were designed with adjustments to the hydrogen peroxide activator quantities. These subsequent experiments were performed at a slightly higher temperature (80 °C) as the product concentrations approached the maximum limits recommended by the supplier. The pH was maintained at 11 for all of the experiments. The selection of peroxide activators and the respective dosage ranges were based on data from the literature,^2,8,9,13,14^ on practical knowledge of process production, and on the results obtained in the initial experiments.

**Table 2:** Chemicals with Fixed Concentrations Used in All Formulations (1 to 17)

Product	(% owf)
Wetting agent	1.0
Chelating agent	0.38
Sodium hydroxide 50%	3.3
Hydrogen peroxide 50%	3.0

**Table 3:** Different Amounts of Hydrogen Peroxide Activators Used in Experiments 1 to 17 at 75 °C and 18 to 28 at 80 °C, along with Berger Whiteness Degree Results

Percent over weight of fabric (owf)					Berger degree	
Triacetin	Peracetic acid	Performic acid	Temperature	Experiment	15 min	30 min
1.12	0	0	75	01	53.9	58.0
			80	19	58.32	58.36
1.80	0	0	75	02	54.9	58.3
0.9	1.0	0	75	03	55.3	56.4
			80	20	59.68	61.09
0.9	0.5	0	75	04	52.5	57.8
0.45	1.0	0	75	05	54.1	56.0
0.45	0.5	0	75	06	54.0	55.7
			80	26	56.34	57.05
0.9	0	1.0	75	07	53.7	56.1
			80	18	56.62	57.00
0.9	0	0.5	75	08	53.4	58.7
0.45	0	1.0	75	09	55.9	57.2
			80	21	56.55	59.82
0.45	0	0.5	75	10	54.0	56.5
			80	27	56.06	57.56
0	1.0	0	75	11	56.0	56.7
			80	22	56.88	60.43
0	0.5	0	75	12	53.6	54.2
			80	24	56.30	58.99
0	0	1.0	75	13	52.3	53.6
0	0	0.5	75	14	52.0	52.8
0	1.0	0.5	75	15	54.5	57.1
			80	23	57.00	58.96
0	0.5	0.5	75	16	52.3	55.2
0	0.5	1.0	75	17	52.5	55.8
0.45	0.5	0.5	80	28	56.83	59.26
0.45	0	0	80	25	56.02	59.63

#### 2.2.2 Chemical Bleaching

The cotton knit substrates were bleached using the exhaustion process in a Kimak AT1-SW dyeing machine equipped with infrared (IR) heating.

Initially, the formulations detailed in Table 3 were prepared. Each chemical was added individually, and the solution was adjusted to the desired volume, according to a bath ratio of 1:10. The chemical bleaching curves are depicted in Figures 3 and 4, where point S marks the start of the chemical dosing process.

**Figure 3:**
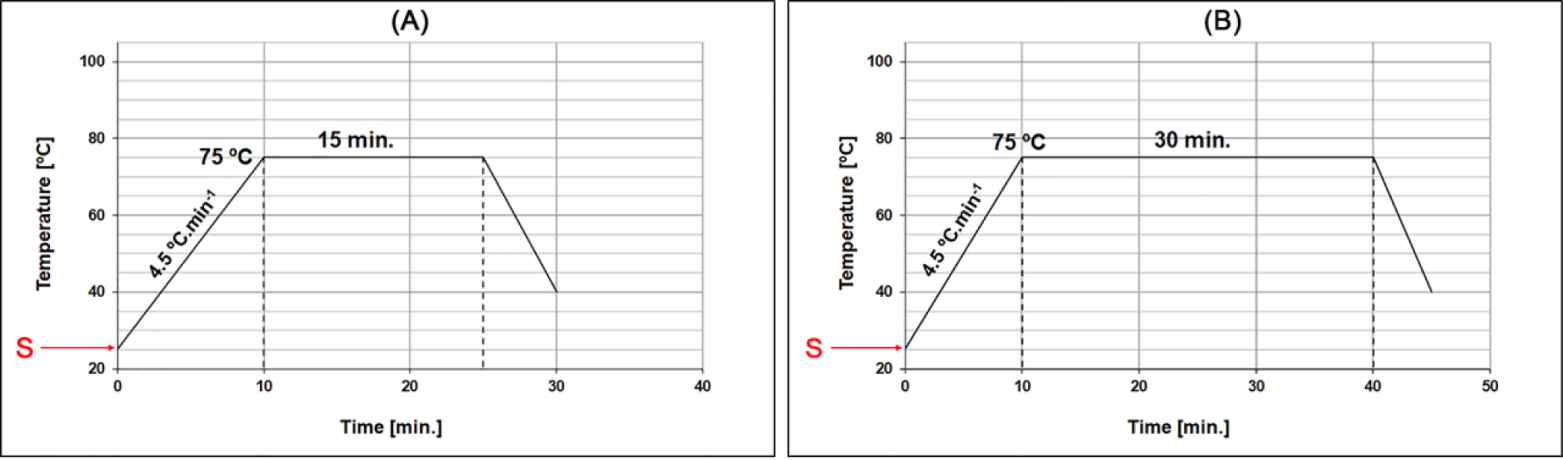
Chemical bleaching diagrams: (A) 15 min and (B) 30 min at 75 °C.

**Figure 4:**
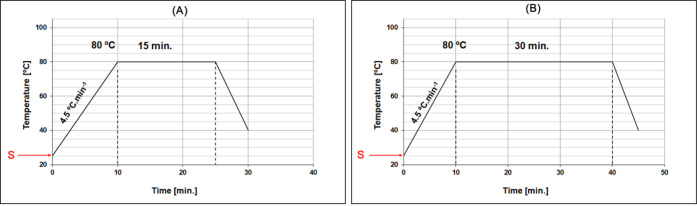
Chemical bleaching diagrams: (A) 15 min and (B) 30 min at 80 °C.

All bleaching tests were conducted using knitted fabrics from the same yarn and weave batches to minimize interference from variations in the origin of the cotton fiber and/or yarn, which could affect the bleaching outcomes. In industrial practice, maintaining consistent batch control is sought but is often challenging due to variations in the cotton batches used by spinning mills, even for yarns of the same title.

No standardized tolerance criterion has been defined by official institutes. However, in industrial practice, a variation of up to 5 points on the CIE whiteness degree scale [D65/10°] is considered acceptable, ensuring no noticeable difference in the final dyed color. In this study, a similar criterion is applied using the WI Berger whiteness scale [D65/10°], as is widely recognized in the industry.

Each bleaching test was performed with 10.0 g of knitted fabric. Considering that the knitted fabric has a weight of 175 g·m^–2^, each 10 g sample measures approximately 23.9 cm × 23.9 cm. Auxiliary chemicals—including wetting agents, Ca and Mg complexing agents, sodium hydroxide (50%), hydrogen peroxide (50%), triacetin, peracetic acid, and performic acid—were weighed and diluted individually at a ratio of 1:10 (chemical to bath). Specifically, 10.0 g of each chemical were weighed and diluted with industrial water to a final volume of 100 mL using a volumetric flask.

#### 2.2.3 Dyeing of Bleached Fabrics

Dichromy, or the use of two dyes, is used in light color formulations to achieve more precise and stable hues. By blending two dyes, formulators can:

Fine-tune the shade: One dye might be too warm or cool, so the second dye balances it.

Improve color fastness: Some dyes help each other resist fading better.

Enhance the brightness or depth: One dye can boost the vibrancy of the other.

This technique is especially useful in delicate or pastel tones, where subtle color shifts matter a lot.

The formulation chosen for dyeing the bleached samples was a light pink color, considered a light/clean hue, which requires a high degree of whiteness. The formulation has a concentration (o.w.f.) of 0.001% C.I. Reactive Yellow 145 and 0.016% C.I. Reactive Red 195. The auxiliary chemicals used in the dyeing of bleached samples were as follows: 30 g·L^–1^ NaCl, 10 g·L^–1^ Na_2_CO_3_, and 1.5 g·L^–1^ ethoxylated C_12_–C_16_ fatty alcohol (dispersing agent). The bath ratio of 1:10 (m/v) with industrial water was used.

Dyeing and washing were carried out in a Kimak “water bath” model AT-HT 2 dyeing machine. The samples were prepared in duplicate and were dyed as per Figure 5, under agitation, starting at 30 °C, heating at a rate of 4.5 °C·min^–1^ up to 60 °C, and kept at that temperature for 20 min. After this time, sodium carbonate was added, and the process was maintained for another 70 min. Subsequently, the equipment was cooled to 40 °C, and the dyed samples went through neutralization, washing, drying, and subsequent color assessment via a spectrophotometer.

**Figure 5:**
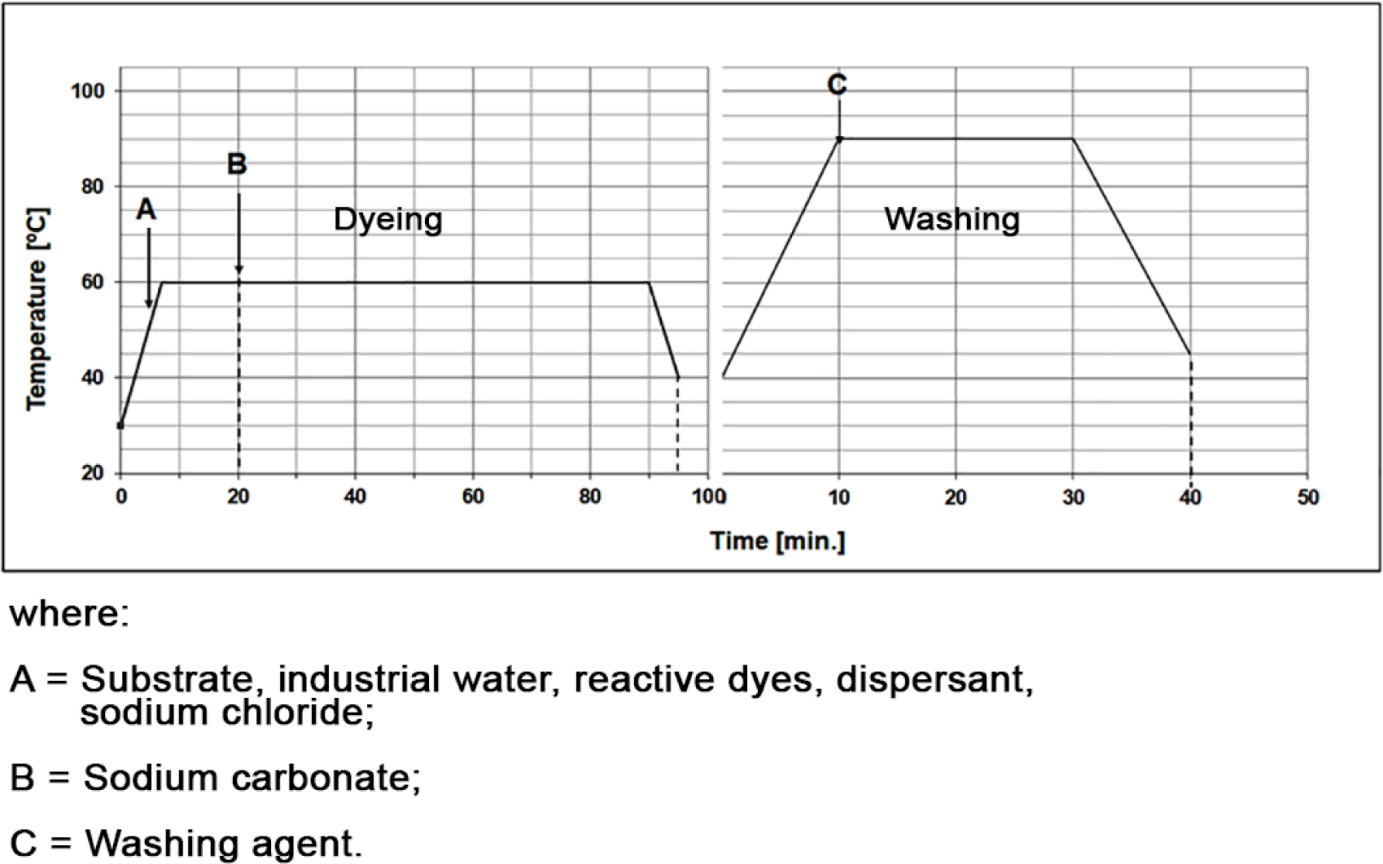
Dyeing diagram.

The neutralization and washing processes aim to remove the remaining electrolytes and hydrolyzed dye that remained in the bath. Neutralization was done by immersing the dyed samples in a solution of citric acid and industrial water with pH = 6.0 −7.0.

In the washing process, the dyed and neutralized samples were subjected to a washing agent in the proportion of 1.2 g·L^–1^ in a ratio of 1:10 (m/v) and heated to 90 °C for 20 min. Afterward, the samples were dried.

### 2.3 Analysis

#### 2.3.1 Whiteness Degree and Color Measurements

Measurements of the whiteness degree of the bleached fabrics, tinctorial strength, and color difference of the dyed fabrics were carried out on an X-rite spectrophotometer coupled to a computer, which uses the Color IQC IMatch software, according to the technical standards: ABNT NBR ISO 105-J01:2008 ^18^ and ABNT NBR ISO 105-J02:2011.^19^

Some important precautions were taken in these tests, such as keeing the samples open in a controlled environment at 23 °C for 24 h to avoid creasing and for acclimatization before the readings.

The samples were folded into four layers of fabric to prevent light transmission. An aperture with an area of 25 mm^2^ and a D65/100 illuminant were used for both bleached and dyed samples.

In this way, the color strength or tinctorial power was assessed using the reflectance spectrophotometer according to AATCC Evaluation Procedure 6–2016 ^20^ based on the Kubelka–Munk equation (eq 1):

1 $$\frac{K}{S}=\frac{{\left(1-R\right)}^{2}}{2R}$$

where *S* is the unit scattering coefficient, *R* is the reflectance, and *K* is the unit absorption coefficient.

The function *K*/*S* is proportional to the dye concentration. The Kubelka–Munk equation is used to relate the coloring power of the dyestuffs.

#### 2.3.2 Weight Loss

The weight loss (wt %) of the three samples that presented the highest Berger whiteness degree (WI_Berger) was calculated. The fabric weight of each respective test specimen was recorded both before and after bleaching at 75 °C, representing the dried sample weight loss. The drying conditions were 100 °C for 1 h. Subsequently, the samples were cooled in a closed desiccator and weighed. Equation 2 was used to calculate the weight loss:

2 $${\mathrm{Wt}}_{\%}=\frac{W_{1}-W_{2}}{W_{1}}$$

where *W*_1_ and *W*_2_ are the weights of the fabric before and after treatment, respectively.

#### 2.3.3 Fabric Hydrophilicity—Water Absorbency Test

Hydrophilicity defines whether the fabric undergoes changes in its water absorption properties after low-temperature bleaching with different peroxide activators. This analysis is important because of the subsequent dyeing process to which the material will be subjected. Ensuring uniform dyeing is a very important parameter to be analyzed.

The test was performed according to AATCC Test Method 79-2007.^21^ The specimen is mounted in a hoop; the fabric should be free from wrinkles, but it should not be too tense either. A buret is placed 9.5 mm above it. A drop of a blue-colored aqueous solution falls on to the specimen, and the time is measured until the drop loses its specular reflection. This duration is termed the wetting time. An average time is calculated for the four specimens.^21^

#### 2.3.4 Fabric Capillarity

The hydrophilicity test using the capillary rise method, known as the capillarity test, was carried out on the bleached samples.

This test consists of marking dry samples of bleached fabric with a stamp, then placing these samples vertically on a support, and immersing 1.0 cm of the end of the fabric in a dye solution (1:10 (m/v)) for a period of 120 s. At the end, the height is measured in centimeters, and the uniformity of the column of solution absorbed by the sample is observed.

The objective of capillary tests is to verify the speed and uniformity of the wetting of the treated samples. The standard height achieved for samples treated at 75 °C is 3.0 cm, and for samples treated at 100 °C, it is 7.0 cm. It is essential that the dye column remain uniform and constant throughout the test.

#### 2.3.5 Scanning Electron Microscopy (SEM)

The surface of selected bleached fabrics was evaluated with a JEOL JSM-6390LV scanning electron microscope at the Central Laboratory of Electronic Microscopy at the UFSC in Florianópolis.

#### 2.3.6 Tensile Properties

Average tensile strength and percent elongation at maximum force for crude and selected bleached cotton samples were measured according to ISO 13934-2:2014.^22^

## 3 Results and Discussion

### 3.1 Colorimetric Whiteness Evaluation

The whiteness degree used as a standard for comparison was 62.94 Berger degrees, a value adopted by the company Brandili for the range of light colors. This degree of whiteness is achieved in the conventional bleaching process at 100 °C for 15 min. However, for medium and dark colors, bleaching is carried out at 75 °C for 15 min, resulting in a whiteness degree of 51.29 Berger degrees.

With the aim of reducing energy consumption and processing time, bleaching experiments were conducted using different combinations of activators (triacetin, peracetic acid, and performic acid) at 75 °C for 15 min, seeking to approach the whiteness degree of light colors while reducing the energy footprint. Subsequently, the experiments were repeated with a processing time of 30 min. Table 3 presents the whiteness degrees of the series of experiments at 75 and 80 °C for 15 and 30 min. Figures S1 and S2 also display all whiteness results.

All experiments showed whiteness degrees lower than the standard, especially those conducted at 75 °C with a 15 min processing time. In experiment 1, with 15 min of processing and using only triacetin as an activator, a whiteness degree of just 53.9 Berger degrees was achieved. Although lower than the standard for light colors, this value is slightly higher than that obtained in the conventional process for medium- and dark-color dyeing. In experiment 2, even with an approximate 61% increase in the percentage of triacetin, the Berger degree values were similar for both processing times, indicating a possible saturation of the peroxide activator.

Overall, combinations with different activators did not result in significant improvements with the 15 min processing time. However, with an increase in processing time to 30 min, the results were better. For example, in experiment 8, which used triacetin (0.9%) and performic acid (1.0%), the highest whiteness degree at 75 °C was achieved, reaching 58.7 Berger degrees.

Based on the best results obtained in experiments 1 to 17, the tests were repeated at 80 °C to further approach the target whiteness degree of 62.94 Berger. By increasing the temperature to 80 °C, a significant improvement in whiteness degrees was observed, as shown in Table 3.

Experiment 20, which used triacetin (0.9%) and peracetic acid (1.0%), achieved the best result among all experiments, with 61.09 Berger degrees after 30 min. This result demonstrates that the combination of activators at higher temperatures favored the bleaching reaction. Considering that peracetic acid is generated during the chemical process, it was observed that the increase in the “pure” activator contributed to improving the whiteness. This result represents 97% of the target value of 62.94 Berger degrees, highlighting the effectiveness of this formulation with a 20 °C reduction compared to the conventional process. These results partially corroborate the findings of Zhou et al. (2021), who obtained satisfactory brightness using 2.2 g·L^–1^ of triacetin at 60 °C for 60 min on cotton mesh. However, the present study demonstrated technical superiority in achieving better brightness levels with a lower triacetin concentration (0.9 g·L^–1^), in a shorter time (30 min) and with a higher temperature (80 °C), reinforcing its potential for industrial application.^16^ These results are also supported by recent literature, as demonstrated by Huang et al., who used a TAML/H_2_O_2_ catalytic system to promote the efficient bleaching of cotton at low temperatures. Like the system tested in this study (triacetin/H_2_O_2_), the TAML system generates highly reactive oxidizing species with satisfactory performance under moderate temperature conditions. Direct comparison indicates that both approaches promote advances toward more sustainable processes with lower thermal impact in the textile chain.^15^ However, although the system of Huang et al. achieves a higher brightness index (72 CIE WI), it requires more time (60 min) and the use of nanostructuring technology for AS dispersion.

Experiments using only peracetic acid, such as experiments 22 and 25, also showed considerable increases in whiteness degrees, achieving 60.43 and 59.63, respectively. In experiment 21, which employed a combination of triacetin (0.45%) and performic acid (1.0%), a good whiteness degree of 59.82 Berger was also achieved.

In experiment 19, compared to experiment 1, it was observed that for the 15 min processing time, there was a considerable improvement in whiteness degree, from 53.9 to 58.32 Berger degrees, indicating the positive effect of increasing the temperature on bleaching. However, with 30 min of processing, the values obtained were very similar. This suggests possible saturation of triacetin, the only activator used in these formulations.

Recent findings also suggest that the efficiency of peroxide-based bleaching systems may be significantly influenced by electrostatic interactions between cotton fibers and bleach components under alkaline conditions.^11^ These interactions become particularly relevant in systems containing triacetin and peracids due to the presence of polar species generated during the reactions, whose activity and adsorption may be affected by the surface charge of the substrate. Therefore, formulation optimizations—such as the inclusion of surfactants or electrolytes—can enhance the bleaching performance by promoting these electrostatic interactions, as observed in this study.

### 3.2 Weight Loss Test

According to ABNT NBR 10591:2008,^23^ the allowable tolerance among the results obtained in the weight loss test is up to 5.0%, or this value can be agreed between the supplier and the customer. In this study, an acceptable tolerance of up to 7.0% was considered, which is the tolerance adopted by Brandili Têxtil.

Table 4 shows that increasing the duration and temperature of the bleaching process results in a higher weight loss.

**Table 4:** Weight Loss

Sample	Experiment	Average weight loss (%)
Crude fabric	-	0.00
Conventional bleaching	-	6.40
75 °C, 30 min	08	2.83
80 °C, 30 min	20	6.01
80 °C, 30 min	22	6.10

Piccoli, de Souza, and de Souza^3^ report the relationship between weight and whiteness in bleaching research using ozone. In this study, it was observed that when the weight of the knitted fabric is lower, a higher degree of whiteness is achieved. Conversely, when the weight of the knitted fabric is higher, the degree of whiteness achieved is lower. This result demonstrates that the mass of cotton fiber involved influences the degree of whiteness.

In another low-temperature bleaching study using sodium chlorite and hexamethylenetetramine, the effect of bleaching temperature on the physicochemical properties of fabrics was also discussed. The study found that the percentage of fabric weight loss increases with temperature, resulting in greater whiteness.^24^

Although the results obtained (Table 4) demonstrate a greater mass loss (6.1%) when bleaching at 80 °C for 30 min, this outcome does not imply unexpected losses for the company, as it falls within the acceptable range. Moreover, this mass loss value remains lower than that obtained through the conventional bleaching method, which recorded a 6.4% loss. Therefore, in terms of both bleaching effectiveness and mass loss, the formulation proposed in experiment 20 appears to be suitable.

### 3.3 Fabric Hydrophilicity—Water Absorbency Test

The evaluation of fabric hydrophilicity aims to determine whether the fabric undergoes changes in its absorption properties after low-temperature bleaching with various peroxide activators. This analysis is crucial for ensuring the fabric’s suitability for the subsequent dyeing process.

Two effective criteria for assessing the bleaching quality are hydrophilicity and capillarity measurements. Hydrophilicity is evaluated using the one-drop absorption method, as detailed in section 2.3.3, while capillarity is measured according to the procedure described in section 2.3.4.

Higher values for these parameters indicate an improved potential for the consistent wetting and water absorption of the fabric after bleaching. Success in these aspects ensures optimal performance, forming a solid foundation for subsequent processes.

However, according to Abdel-Halim, the bleaching process can be compromised when carried out at low temperatures due to the reduced removal of noncellulosic materials from the fabric, which consequently reduces water absorption. The resulting material ends up with a dark color and a hydrophobic character.^24^ Therefore, the results obtained in this study confirmed a direct relationship between the temperature and the hydrophilicity of the treated samples. Samples treated at 75 °C showed a hydrophilicity of 2.3 min, whereas for samples treated at 80 °C for 15 and 30 min, it was 40 and 26 s, respectively. These results also indicate that an increasing processing time affects the achieved hydrophilicity values. According to the literature, the raw fabric takes more than 10 min to absorb a water drop.^25^

Regarding the results obtained in the capillarity test, for samples treated at 75 °C, for both 15 and 30 min, a height of 1 cm was reached. Figure 6 displays the results obtained for bleaching at 80 °C.

**Figure 6:**
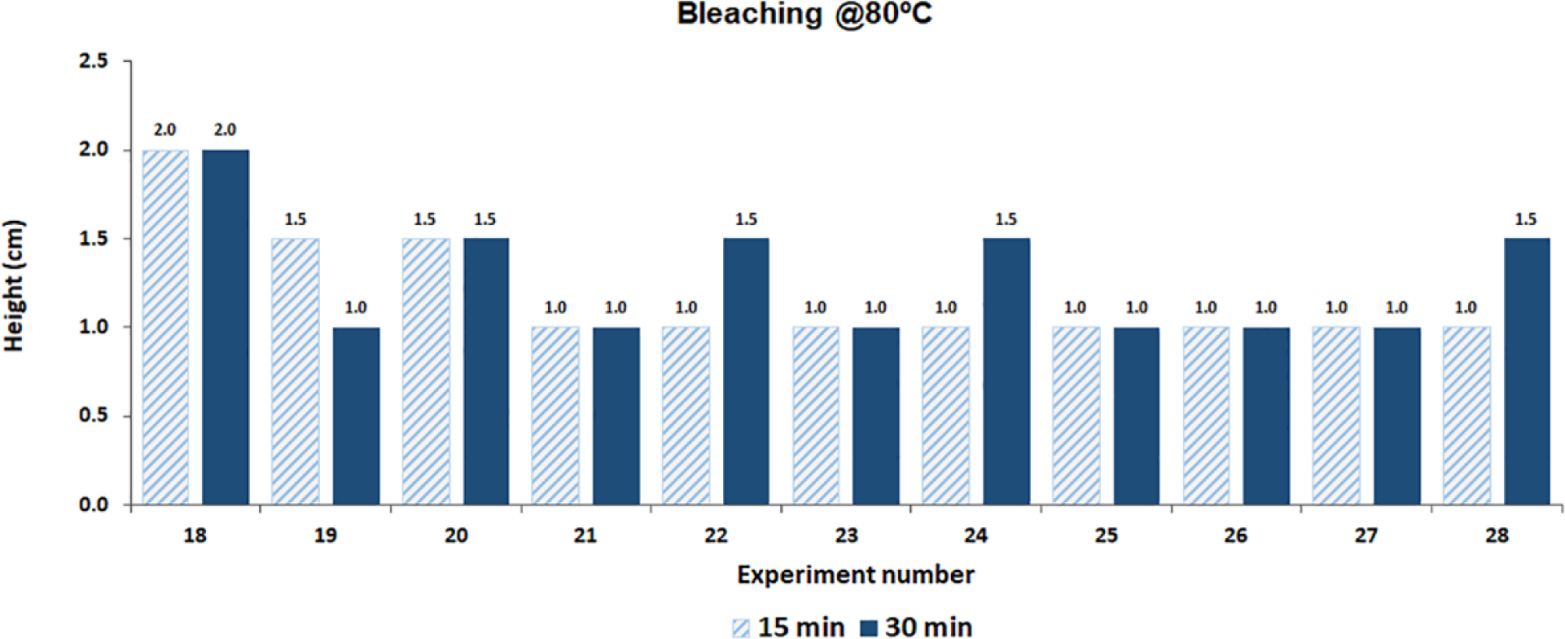
Capillarity of samples.

In this case, the best results were obtained from sample 18, treated at 80 °C for 30 min, where the droplet spread occurred in 26 s, and the capillarity height was 2.0 cm. In their study, the author cites an instantaneous hydrophilicity of the droplet and capillarity of 4.5 and 4.0 cm for bleaching at 75 and 95 °C, respectively.^25^ Although the observed values are lower than those of conventional processes, they are within acceptable limits for industrial applications.

### 3.4 Dyeing of Bleached Fabrics

After the bleaching process was completed, the samples displaying the highest degree of whiteness at each temperature and time were chosen for the subsequent dyeing process using a light pink shade. This particular color was selected because it facilitates the assessment of the bleaching process’s quality. Being categorized as a “light/clean” color, its excellent reproducibility allows for the verification of effective bleaching. Currently, conventional bleaching at 100 °C is employed for this color.

The dichromy was achieved by mixing two reactive dyes for dyeing the bleached samples: C.I. Reactive Yellow 145 (0.001% owf) and C.I. Reactive Red 195 (0.016% owf), as detailed in section 2.1.1.

Industrial color production applications commonly utilize the CMC color difference, a pass/fail method in which a single numerical tolerance can be established and used to make acceptability decisions. The CMC color difference formula is based on the colorimetric principles of the CIE 1976 system. CMC color difference (Δ*E* CMC-l:c), a modification of CIE L*C*h color difference, has proven to be a useful measure of the commercial acceptability of colored products.^26^ The parameters l and c, typically expressed as CMC­(l:c), represent the values for acceptability (CMC-2:1 and perceptibility (CMC-1:1), respectively.^27,28^

The values obtained for color difference (Δ*E* CMC1:2), tinctorial power, and *K*/*S* sum values for selected dyed samples are presented in Table 5. The sample from the conventional bleaching was used as the standard.

**Table 5:** Color Difference Values (Δ*E* CMC1:2), Tinctorial Power, and *K*/*S* Sum Values for Standard and Selected Dyed Samples

Sample	ΔE CMC1:2	*K*/*S* sum value	Tinctorial power (%)
Standard	-	0.7015	100.00
08	0.96	0.7511	107.07
20	0.53	0.7242	103.23
22	0.62	0.7360	105.05

From the results, it can be seen that all of the samples bleached with activators have excellent color properties after dyeing. Snapshots of the selected dyed samples presented in Figure 7 show that bleaching with activators is suitable as a pretreatment for light colors and results in no interference or yellowing of the samples.

**Figure 7:**
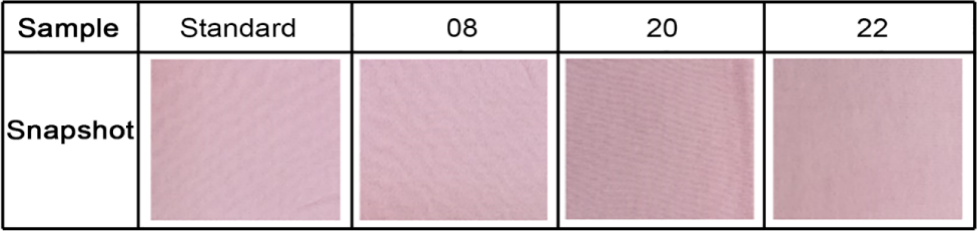
Snapshots of the dyed samples.

### 3.5 Scanning Electron Microscopy (SEM)

Morphological evaluations of the samples that achieved the best degrees of whiteness were carried out using scanning electron microscopy, as shown in Figure 8 with magnifications of 50×, 1000×, and 4000×, along with the raw substrate. The images reveal that the samples have smooth surfaces with no signs of damage due to the chemical action of the activators.

**Figure 8:**
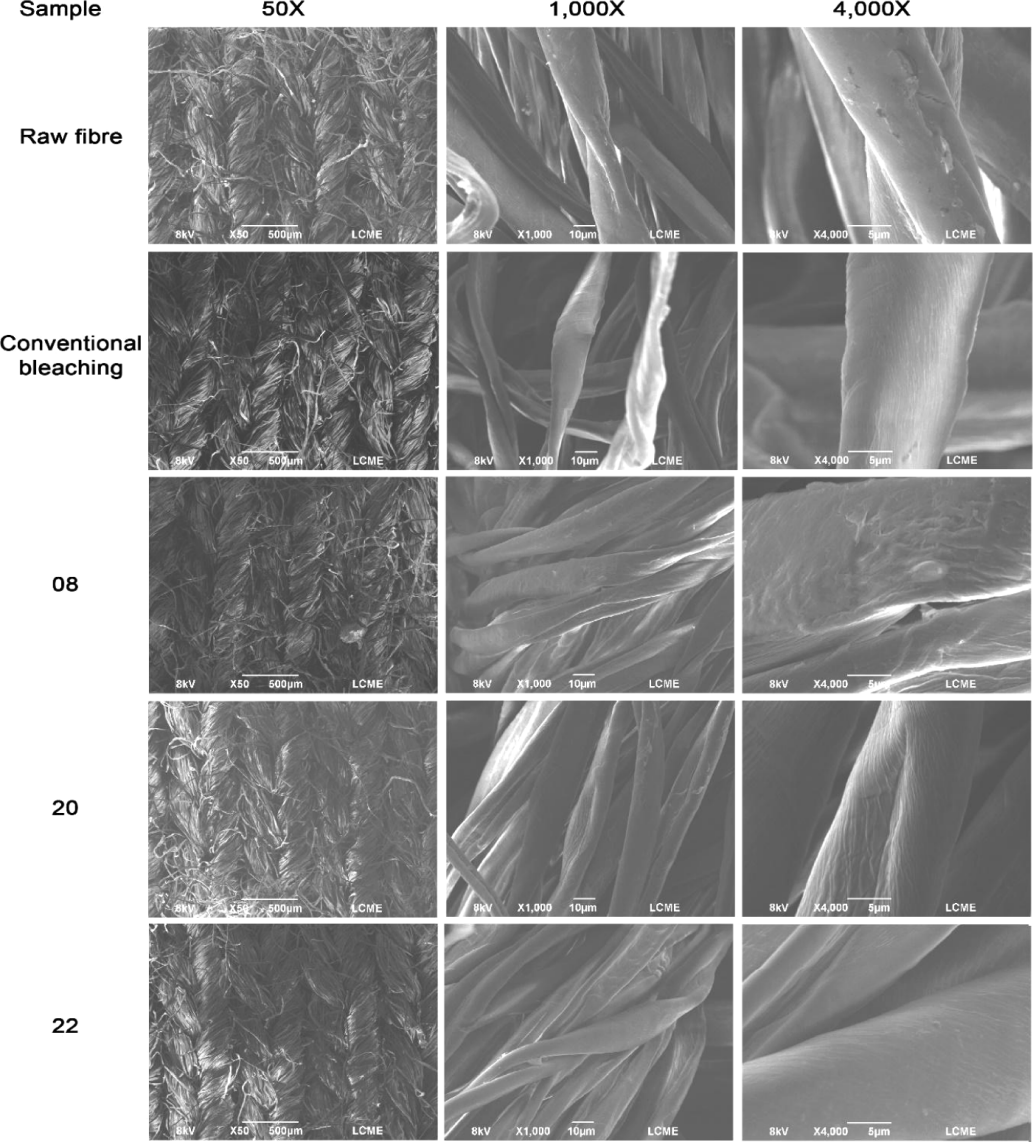
SEM images of raw and bleached fibers.

### 3.6 Tensile Properties

The literature reports that the testing results for the tensile strength of cotton fabric after conventional bleaching are 303 N.^29^ However, such values are strongly dependent on yarn and fabric construction properties.^30^ Table 6 displays the tensile properties of selected bleached samples by comparing them with the crude fabric and the sample subjected to conventional bleaching.

**Table 6:** Tensile Properties of Cotton Samples

Sample	Maximum force (N)	Elongation at break (%)
Crude fabric	431.02 ± 26.50	12.67
Conventional bleaching	412.32 ± 23.40	10.55
08	419.46 ± 25.79	18.33
20	414.36 ± 25.48	22.57

The results indicate, as expected, that the crude fabric exhibits the highest maximum force (431.02 ± 26.50 N) compared with the other treatments, suggesting that the bleaching process can reduce the mechanical strength of the fabric. Furthermore, crude fabric tends to be more rigid, which explains its lower elongation at break (12.67%) compared to that of the treated samples.

Among the treatments, conventional bleaching results in an even greater reduction in elongation (10.55%), suggesting that this treatment makes the fabric more fragile or less elastic, as evidenced by the lower maximum breaking force (412.32 ± 23.40 N). Similarly, treatments “08” (419.46 ± 25.79 N) and “20” (414.36 ± 25.48 N) show intermediate values of maximum force, indicating that these methods may better preserve the fabric’s strength compared to the conventional method. However, considering the standard deviations of the mean values, the differences in the maximum force can be deemed negligible.

Regarding elongation at break, treatments “08” (18.33%) and “20” (22.57%) significantly increased the elongation, indicating that these treatments can enhance the fabric’s flexibility and its ability to withstand deformation before breaking. Therefore, these treatments can be considered interesting alternatives to conventional bleaching, especially if the goal is to improve elasticity without significantly compromising mechanical strength.

## 4 Conclusions

This study reveals that the use of hydrogen peroxide activators, such as triacetin and peracetic acid, in the bleaching process of cotton fabric samples prior to dyeing is an efficient alternative to traditional methods. The application of these activators allows for achieving high levels of whiteness, while significantly reducing the energy consumption and the time required for the process, when compared to conventional techniques.

Although the hydrophilicity and capillarity of the fabric presented values slightly lower than those reported in the literature, this difference did not compromise the quality of the dyeing process, especially for light colors, resulting in a satisfactory performance. In addition, the mechanical properties and integrity of the fabric were preserved, demonstrating the effectiveness of the method in maintaining the essential characteristics for subsequent dyeing. By using 0.9 g/L of triacetin and 1.0 g/L of peracetic acid at 80 °C for 30 min, a whiteness degree of 61.1 Berger was achieved. This represents a significant reduction in the consumption of the activators compared to a previous study in the literature.

From an economic point of view, the process demonstrated substantial advantages with a 20% reduction in energy consumption and processing time. These operational gains, together with the maintenance of the quality of the final product, indicate the viability of the method in terms of cost–benefit.

Therefore, the proposed bleaching approach shows great promise for industrial applications regardless of the color range to be used. Furthermore, it contributes significantly to the transition toward more sustainable industrial processes, with lower environmental impacts and greater production efficiency.

## Supplementary Material



## Notes

Berger whiteness degrees—experiments at 75 °C (Figure S1); Berger whiteness degrees—experiments at 0 °C (Figure S2) (PDF)

## Abbreviations

AATCC: American Association of Textile Chemists and Colorists
ABNT: Associação Brasileira de Normas Técnicas
CIE: International Commission on Illumination
CMC (l:c): Color Measurement Committee
DOE: Design of Experiments
GT: Glycerol triacetate
HDEPA: 1-Hydroxyethane-1,1-diphosphonic acid
ISO: International Organization for Standardization
K/S: Kubelka–Munk Equation
NBR: Norma Brasileira Regulamentadora
NOBS: Sodium nonanoyloxybenzenesulfonate
PAA: Peracetic acids
SEM: Scanning Electron Microscopy
TAED: Tetraacetylethylenediamine
TH: Tetraacetylhydrazine
UFSC: Universidade Federal de Santa Catarina
WI: Whiteness Index
owf: Over Weight of Fabric
