# Supplementary material for: Performance of Low-Temperature Bleaching Techniques for Cotton Fabrics Using Hydrogen Peroxide Activators
Source: ACS Omega. 2025 Jun 27;10(26):28167–77. doi: 10.1021/acsomega.5c02605 (PMC12242642; doi:10.1021/acsomega.5c02605)

Performance of Low-Temperature Bleaching Techniques for Cotton Fabrics Using Hydrogen Peroxide Activators

Letícia Küster, Bruna Porto, Catia Rosana Lange de Aguiar, and Miguel Angelo Granato*

Postgraduate Program in Textile Engineering, Federal University of Santa Catarina, Blumenau Campus, Blumenau, Santa Catarina, Brazil. João Pessoa St., 2750, Velha, 89036-256 – Blumenau, SC, Brazil

Supplementary Information

Figure S1. Berger whiteness degrees – Experiments @ 75ºC

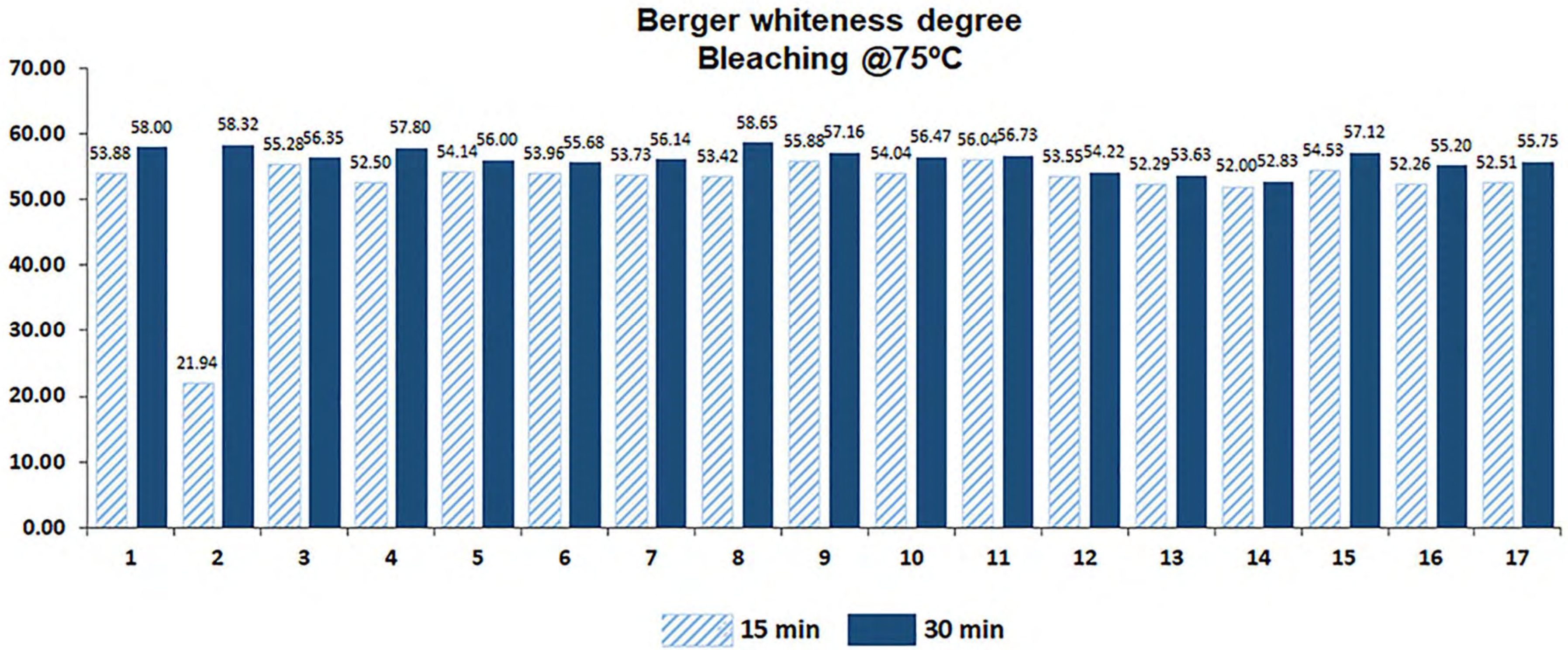

Figure S2. Berger whiteness degrees – Experiments @ 80ºC

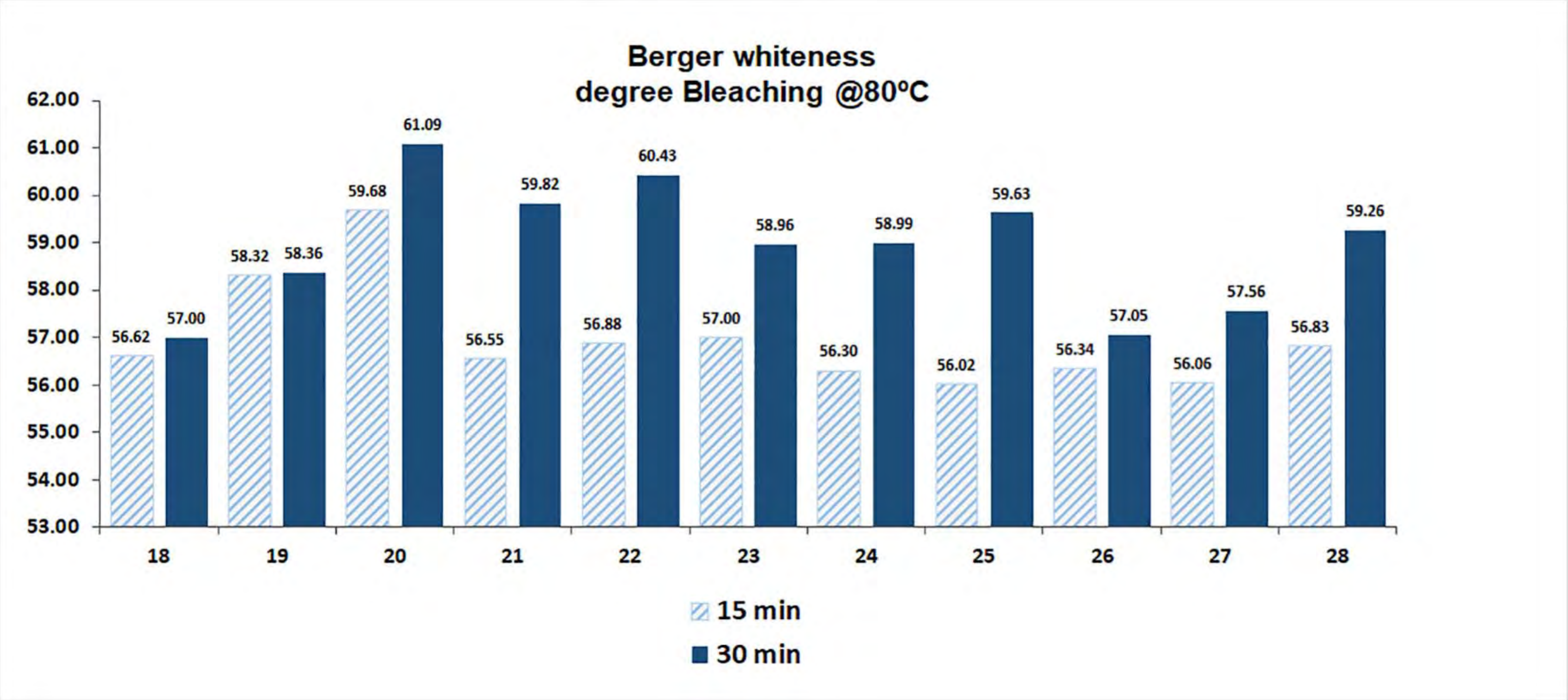

Supplement: Supplementary file 1 [file ao5c02605_si_001.pdf]
